# Are Ethnic and Gender Specific Equations Needed to Derive Fat Free Mass from Bioelectrical Impedance in Children of South Asian, Black African-Caribbean and White European Origin? Results of the Assessment of Body Composition in Children Study

**DOI:** 10.1371/journal.pone.0076426

**Published:** 2013-10-18

**Authors:** Claire M. Nightingale, Alicja R. Rudnicka, Christopher G. Owen, Angela S. Donin, Sian L. Newton, Cheryl A. Furness, Emma L. Howard, Rachel D. Gillings, Jonathan C. K. Wells, Derek G. Cook, Peter H. Whincup

**Affiliations:** 1 Division of Population Health Sciences and Education, St George's, University of London, London, United Kingdom; 2 Childhood Nutrition Research Centre, UCL Institute of Child Health, London, United Kingdom; CUNY, United States of America

## Abstract

**Background:**

Bioelectrical impedance analysis (BIA) is a potentially valuable method for assessing lean mass and body fat levels in children from different ethnic groups. We examined the need for ethnic- and gender-specific equations for estimating fat free mass (FFM) from BIA in children from different ethnic groups and examined their effects on the assessment of ethnic differences in body fat.

**Methods:**

Cross-sectional study of children aged 8–10 years in London Primary schools including 325 South Asians, 250 black African-Caribbeans and 289 white Europeans with measurements of height, weight and arm-leg impedance (Z; Bodystat 1500). Total body water was estimated from deuterium dilution and converted to FFM. Multilevel models were used to derive three types of equation {A: FFM = linear combination(height+weight+Z); B: FFM = linear combination(height^2^/Z); C: FFM = linear combination(height^2^/Z+weight)}.

**Results:**

Ethnicity and gender were important predictors of FFM and improved model fit in all equations. The models of best fit were ethnicity and gender specific versions of equation A, followed by equation C; these provided accurate assessments of ethnic differences in FFM and FM. In contrast, the use of generic equations led to underestimation of both the negative South Asian-white European FFM difference and the positive black African-Caribbean-white European FFM difference (by 0.53 kg and by 0.73 kg respectively for equation A). The use of generic equations underestimated the positive South Asian-white European difference in fat mass (FM) and overestimated the positive black African-Caribbean-white European difference in FM (by 4.7% and 10.1% respectively for equation A). Consistent results were observed when the equations were applied to a large external data set.

**Conclusions:**

Ethnic- and gender-specific equations for predicting FFM from BIA provide better estimates of ethnic differences in FFM and FM in children, while generic equations can misrepresent these ethnic differences.

## Introduction

Obesity prevalence has risen in the UK and worldwide [1], [2], with important long-term consequences for risks of type 2 diabetes (T2D) and cardiovascular disease (CVD) [1], [3]. In the UK, the consequences may be particularly important among children of South Asian and black African-Caribbean origin, with their high long-term risks of T2D and CVD [4]–[6] originating in childhood [7], [8] and increased metabolic sensitivity to adiposity particularly among South Asians [9], [10]. Accurate measurement of body fat levels among children of different ethnic groups is therefore important. However, body mass index (BMI), the most widely used obesity marker in children [11], underestimates body fat levels among South Asians [12], [13] and overestimates body fat levels among black African-Caribbeans [13]. Other valid approaches to body fat measurement in children from different ethnic groups are therefore needed. Ideally such methods should also provide accurate information on lean mass, which may also differ between ethnic groups and influence type 2 diabetes risks [14], [15].

Bioelectrical impedance analysis (BIA) is a method for deriving fat free mass (FFM), and indirectly fat mass (FM), from electrical resistance [16] and may provide valid body fat measurements in children of different ethnic groups [13]. However, the validity of BIA depends on the validity of the equation(s) used to derive FFM [17]. Several equations have been validated, generally deriving FFM from linear regression equations including weight, height and impedance terms [18] or from equations including height^2^/impedance [19]; the latter method assumes that the body has cylindrical proportions [16]. However, these equations have largely been validated in white European or American populations and there is little information on their validity in children from different ethnic groups, particularly before puberty, though a recent study in adolescents suggested that there were marked ethnic differences in optimal prediction equations for FFM [20]. We therefore designed a new study, the Assessment of Body Composition in Children (ABCC) Study to examine whether equations for deriving FFM from BIA (measured between the arm and leg using the Bodystat 1500 body composition analyser) need to be ethnic- and gender-specific for use in children of South Asian, black African-Caribbean and white European origin. We compared the impact of using generic and ethnic- and gender-specific equations on the estimation of ethnic differences in FFM. Because of the importance of the accurate assessment of ethnic differences in body fat, we also examined the assessment of FM (calculated as weight minus FFM) and fat mass index (FM(kg)/height(m)^5^). We examined these issues both in the ABCC Study and in an external dataset based on our earlier Child Heart and health Study in England (CHASE).

## Materials and Methods

### Ethics statement

Ethical approval was obtained from the National Research Ethics Service Committee London – Bloomsbury. Parents/guardians were sent invitation letters, translated where necessary; informed written consent was obtained from parents/guardians for all participants.

### Study design

The Assessment of Body Composition in Children (ABCC) Study was a cross-sectional study which aimed to calibrate BIA against the measurement of total body water (TBW) (using deuterium dilution) in London primary school children of South Asian, black African-Caribbean and white European origin. Information on all London state primary schools and pupil ethnicity was provided by the UK Government Department for Education. Schools with high proportions of pupils of Indian, Pakistani, Bangladeshi, black African, black Caribbean and white European ethnic origin were separately identified and a stratified random sample of 24 schools selected to include balanced numbers of South Asian children (including Indian, Pakistani and Bangladeshi), black African-Caribbean children (including black African and black Caribbeans) and white European children (including white British). Schools which did not agree to participate were replaced with schools of a similar ethnic composition.

### Physical assessments and ethnicity

All assessments were carried out between September 2011 and January 2012 by a team of three Research Assistants, trained in all measurement techniques at the outset and reviewed during the study. The Research Assistants rotated roles and each made approximately one-third of measurements of children in each ethnic group in order to minimise bias in ethnic group comparisons. They measured height using a portable stadiometer (Chasmors Ltd., London, UK), weight using a Tanita MA-418-BC body composition analyser (Tanita Inc., Tokyo, Japan) and skinfold thickness at biceps, triceps, subscapular and suprailiac locations using a Holtain skinfold caliper (Holtain Ltd., Crymych, UK). Two consecutive measurements of arm-leg impedance were made using the Bodystat 1500 analyser (Bodystat Ltd, Isle of Man, UK) on the right hand side of the body with the child resting supine; analyses used the average of the two readings. Pubertal status was assessed by the Research Assistant using the Tanner breast development scoring system in girls with the participant wearing light clothing [21]; boys did not undergo pubertal assessment because of their later entry to puberty [22], [23]. Ethnicity was based on parentally defined ethnicity of both parents (available for 79.9% of participants) or on parentally defined ethnicity of the child (available for a further 19.5% of participants). In the remainder (0.6% of participants) ethnicity was defined using information on parental and grandparental places of birth cross-checked with the ethnic appearance of the child at examination.

### Deuterium dilution study

Deuterium dilution was used as a gold standard reference for TBW measurement [24]. Deuterium oxide dosages used 99.8% purity deuterium oxide (CK Gas Products Ltd., Ibstock, UK) and were weighed using scales with accuracy to 0.01 grams. The exact dose amounts of deuterium oxide and filtered water were recorded for all participants and a sample of the dose was analysed. Saliva samples for deuterium measurement were obtained at baseline and 4.5 hours after the participants received their deuterium oxide dose; participants avoided food and drink for at least 30 minutes before each sample. All fluid consumption between the deuterium dose and the second saliva sample was documented. Deuterium concentrations in each saliva sample and each individual deuterium dose were measured by isotope-ratio mass spectrometry (Iso-Analytical Ltd, Crewe, UK) using continuous-flow isotope ratio mass spectrometry. TBW was calculated incorporating a correction for the exchange of deuterium with non-aqueous hydrogen [25] and adjusting for fluid intake during the equilibrium period. FFM was calculated from TBW using assumed hydration of lean tissue [26]; FM was calculated as the difference between body weight and FFM.

### External dataset

The Child Heart and health Study in England (CHASE), a study of the health of 5887 9–10 year-old British school children including balanced numbers of South Asian, black African-Caribbean and white European origin, in which standardized measurements of height, weight and biceps, triceps, subscapular and suprailiac skinfold thicknesses were made using a similar protocol to that in the ABCC Study [13], provided an independent external dataset. A single arm-leg impedance measurement was made using the Bodystat 1500 analyser; no deuterium dilution measurements were made. Skinfold thickness measurements were used to provide an independent marker of adiposity.

### Statistical methods

A sample size with 250 subjects in each ethnic group was based on the ability to detect at least a 15% difference in the regression slope relating height^2^/impedance and TBW. A study of this size would also enable detection of approximately 0.5 SD difference in the intercept of the regression line relating height^2^/impedance and TBW with 90% power and a 5% type I error rate. Statistical analyses were carried out using STATA/SE software (Stata/SE 12 for Windows, StataCorp LP, College Station, TX, USA). Multilevel linear models were used to produce adjusted means with school fitted as a random effect to allow for clustering of children within schools. Likelihood ratio tests were used to test for gender differences and heterogeneity in ethnicity. Variables were inspected for normality using normal probability plots of the raw variable and the residuals from the model and were log transformed where appropriate. FM, FMI and sum of skinfolds index were all log transformed in analyses of ethnic differences.

Equations were generated to predict FFM from BIA using multilevel linear models. Using FFM from deuterium dilution as the dependent variable, we fitted three types of general equations used in earlier reports:[18] [27] [28]

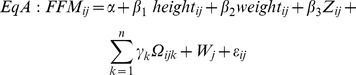


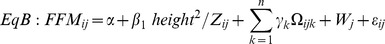


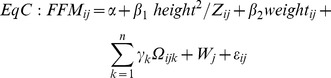



These models refer to the ith individual in the jth school, where Z is impedance, W_j_ is the random effect for school, ε_ij_ is the individual level error and 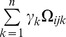 may include main effects for ethnicity and gender and interactions between terms. In the simplest form of each type of model (referred to as generic because it is applied in the same form to all ethnic groups) there are no additional parameters and 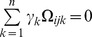.

For each type of model we tested for differences in intercepts and slopes by ethnicity and gender. The goodness of fit of models were compared using likelihood ratio tests (when models were nested) and the proportion of variance explained by covariates. The Akaike information criteria (AIC) were used for comparing non-nested models. The AIC is based on the log likelihood with a penalty based on number of parameters in the model; in a series of models the model of best fit would have the lowest AIC [29]. The proportion of variance explained by covariates in a mixed effects model is a measure of goodness of fit but does not take into account the number of parameters in the model [30]. The model of best fit within each equation type and the corresponding generic equation (with no terms for ethnicity or gender) were selected for comparisons of ethnic differences in FFM, FM and FMI. Since FM and sum of skinfolds were highly correlated with height, height standardized indices were derived by dividing FM and sum of skinfolds by height^5^ and height^3^ respectively to remove the correlation with height [13]. Ethnic differences in sum of skinfolds index were adjusted for small differences between observers; such adjustments were not needed for FFM, FM or FMI. Bland-Altman plots [31] were used to examine bias in prediction of FFM by ethnicity for each selected equation. If there is no prediction bias, the plot should show horizontal regression lines intercepting the vertical axis at zero for all ethnic groups.

## Results

In all, 1352 children of white European, South Asian origin and black African-Caribbean were invited and 864 (64%) took part in the study and had complete body composition data. These included 289 white Europeans, 325 South Asians and 250 black African-Caribbeans (response rates 72%, 64% and 57% respectively) with a mean age of 9.2 (range 8.0–10.6) years. There were marked gender and ethnic differences in body size and composition which are summarised in supporting information file Table S1.

### Deriving equations for fat free mass (FFM) from BIA

Three types of equation were considered when developing equations for deriving FFM, as described in the statistical methods section. FFM was fitted as the outcome variable in Tables 1, 2 and 3, which contained different sets of predictor variables.

**Table 1:** Coefficients from regression models deriving equations for estimating fat free mass by fitting height, weight and impedance as separate variables in the model and adding interaction terms for ethnicity and gender.

	Model A1			Model A2			Model A3			Model A4			Model A5		
Coefficient	β	(95% CI)	p	β	(95% CI)	p	β	(95% CI)	p	β	(95% CI)	p	β	(95% CI)	p
Constant	−6.785	(−2.227, 2.227)	<0.0001	−6.353	(−8.423, −4.282)	<0.0001	−3.920	(−7.755, −0.085)	0.05	−3.919	(−6.551, −1.287)	0.004	−4.334	(−7.756, −0.912)	0.01
Ethnic group BAC				0.909	(0.672, 1.146)	<0.0001	−3.902	(−9.188, 1.384)	0.15	−1.620	(−4.142, 0.902)	0.21	−1.550	(−4.073, 0.973)	0.23
Ethnic group SA				−0.520	(−0.748, −0.292)	<0.0001	−2.398	(−7.358, 2.562)	0.34	−3.925	(−6.512, −1.338)	0.003	−3.800	(−6.393, −1.206)	0.004
Sex (female)				−0.514	(−0.678, −0.349)	<0.0001	−0.446	(−0.729, −0.164)	0.002	−0.512	(−0.672, −0.353)	<0.001	−0.094	(−4.121, 3.933)	0.96
BAC×Sex							−0.336	(−0.746, 0.074)	0.11						
SA×Sex							0.075	(−0.307, 0.457)	0.70						
Height (cm)	0.246	(−0.017, 0.017)	<0.0001	0.230	(0.214, 0.245)	<0.0001	0.231	(0.202, 0.260)	<0.0001	0.230	(0.214, 0.245)	<0.001	0.238	(0.214, 0.262)	<0.0001
BAC×HT							0.016	(−0.025, 0.056)	0.44						
SA×HT							−0.013	(−0.051, 0.024)	0.49						
Weight (kg)	0.215	(−0.015, 0.015)	<0.0001	0.225	(0.210, 0.239)	<0.0001	0.200	(0.170, 0.229)	<0.0001	0.201	(0.177, 0.225)	<0.001	0.191	(0.162, 0.220)	<0.0001
BAC×WT							0.044	(0.007, 0.080)	0.02	0.048	(0.021, 0.075)	<0.001	0.047	(0.020, 0.075)	0.001
SA×WT							0.013	(−0.025, 0.051)	0.50	0.005	(−0.024, 0.034)	0.73	0.005	(−0.023, 0.034)	0.72
Z (ohms)	−0.015	(−0.001, 0.001)	<0.0001	−0.012	(−0.013, −0.011)	<0.0001	−0.015	(−0.017, −0.013)	<0.0001	−0.015	(−0.017, −0.013)	<0.001	−0.015	(−0.018, −0.013)	<0.0001
BAC×Z							0.002	(−0.001, 0.005)	0.20	0.001	(−0.002, 0.004)	0.38	0.001	(−0.002, 0.004)	0.41
SA×Z							0.004	(0.002, 0.007)	0.002	0.004	(0.002, 0.007)	0.001	0.004	(0.002, 0.007)	0.002
Sex×HT													−0.013	(−0.044, 0.018)	0.41
Sex×WT													0.017	(−0.011, 0.045)	0.23
Sex×Z													0.001	(−0.001, 0.003)	0.30
Akaike information criterion		2889.5			2755.2			2715.2			2714.2			2718.4	
Proportion variance explained		0.921			0.932			0.936			0.935			0.935	

Abbreviations: CI, confidence interval; BAC, Black African-Caribbean; SA, South Asian; HT, height; WT, weight; Z, impedance.

All models are adjusted for random effect of school.

**Table 2:** Coefficients from regression models deriving equations for estimating fat free mass by fitting height^2^/impedance as a variable in the model and adding interaction terms for ethnicity and gender.

	Model B1			Model B2			Model B3			Model B4		
Coefficient	β	(95% CI)	p	β	(95% CI)	p	β	(95% CI)	p	β	(95% CI)	p
Constant	3.947	(−0.703, 0.703)	<0.0001	3.995	(3.200, 4.790)	<0.0001	5.548	(4.031, 7.065)	<0.0001	4.217	(2.152, 6.282)	<0.0001
Ethnic group BAC				1.542	(1.161, 1.923)	<0.0001	2.468	(0.773, 4.163)	0.004	5.237	(2.588, 7.886)	0.0001
Ethnic group SA				0.395	(0.042, 0.749)	0.03	−1.399	(−3.141, 0.343)	0.12	−0.119	(−2.887, 2.648)	0.93
Sex (female)				−0.036	(−0.299, 0.228)	0.79	−2.559	(−3.848, −1.271)	0.0001	−0.303	(−2.956, 2.350)	0.82
BAC×Sex										−4.738	(−8.202, −1.274)	0.01
SA×Sex										−2.331	(−5.922, 1.261)	0.20
HT^2^/Z (cm^2^/ohms)	0.738	(−0.025, 0.025)	<0.0001	0.713	(0.687, 0.739)	<0.0001	0.657	(0.602, 0.711)	<0.0001	0.704	(0.631, 0.776)	<0.0001
BAC×HT2/Z							−0.032	(−0.093, 0.028)	0.29	−0.122	(−0.213, −0.031)	0.01
SA×HT^2^/Z							0.073	(0.005, 0.140)	0.03	0.024	(−0.079, 0.127)	0.65
Sex×HT^2^/Z							0.096	(0.048, 0.143)	<0.0001	0.013	(−0.085, 0.111)	0.80
BAC×Sex×HT^2^/Z										0.158	(0.035, 0.281)	0.01
SA×Sex×HT^2^/Z										0.094	(−0.044, 0.232)	0.18
Akaike information criterion		3682.7			3626.5			3603.8			3602.8	
Proportion variance explained		0.795			0.810			0.816			0.818	

Abbreviations: CI, confidence interval; BAC, Black African-Caribbean; SA, South Asian; HT^2^/Z, height^2^/impedance.

All models are adjusted for random effect of school.

**Table 3:** Coefficients from regression models deriving equations for estimating fat free mass by fitting height^2^/impedance and weight as variables in the model and adding interaction terms for ethnicity and gender.

	Model C1			Model C2			Model C3			Model C4		
Coefficient	β	(95% CI)	p	β	(95% CI)	p	β	(95% CI)	p	β	(95% CI)	p
Constant	3.756	(3.242, 4.271)	<0.0001	4.991	(4.449, 5.533)	<0.0001	4.926	(3.914, 5.937)	<0.0001	4.138	(2.777, 5.499)	<0.0001
Ethnic group BAC				0.967	(0.707, 1.227)	<0.0001	2.146	(1.019, 3.272)	0.0002	4.238	(2.492, 5.983)	<0.0001
Ethnic group SA				−0.527	(−0.773, −0.281)	<0.0001	0.012	(−1.150, 1.175)	0.98	1.036	(−0.790, 2.861)	0.27
Sex (female)				−0.559	(−0.738, −0.380)	<0.0001	−1.773	(−2.630, −0.916)	<0.0001	−0.393	(−2.138, 1.352)	0.66
BAC×Sex										−3.509	(−5.789, −1.230)	0.003
SA×Sex										−2.110	(−4.472, 0.253)	0.08
HT^2^/Z (cm^2^/ohms)	0.430	(−0.026, 0.026)	<0.0001	0.378	(0.351, 0.405)	<0.0001	0.419	(0.366, 0.471)	<0.0001	0.461	(0.401, 0.520)	<0.0001
Weight (kg)	0.247	(−0.016, 0.016)	<0.0001	0.257	(0.242, 0.273)	<0.0001	0.226	(0.191, 0.260)	<0.0001	0.214	(0.183, 0.245)	<0.0001
BAC×HT^2^/Z							−0.151	(−0.210, −0.092)	<0.0001	−0.216	(−0.290, −0.142)	<0.0001
SA×HT^2^/Z							−0.026	(−0.096, 0.044)	0.47	−0.063	(−0.150, 0.024)	0.16
Sex×HT^2^/Z							0.076	(0.028, 0.124)	0.002	−0.003	(−0.067, 0.061)	0.93
BAC×WT							0.086	(0.048, 0.124)	<0.0001	0.088	(0.049, 0.126)	<0.0001
SA×WT							0.009	(−0.032, 0.050)	0.68	0.004	(−0.037, 0.046)	0.84
Sex×WT							−0.023	(−0.052, 0.007)	0.13			
BAC×Sex×HT^2^/Z										0.107	(0.026, 0.188)	0.01
SA×Sex×HT^2^/Z										0.094	(0.003, 0.185)	0.04
Akaike information criterion		3063.3			2936.2			2902.7			2888.3	
Proportion variance explained		0.902			0.915			0.920			0.921	

Abbreviations: CI, confidence interval; BAC, Black African-Caribbean; SA, South Asian; HT^2^/Z, height^2^/impedance; WT, weight.

All models are adjusted for random effect of school.

#### Type A equation: FFM = linear combination (height+weight+impedance(Z))

Type A model coefficients are shown in Table 1. A generic model containing height, weight and impedance (but not ethnicity and gender) was fitted (model A1); intercept (main effect) terms for ethnicity and gender were then added (model A2). These were statistically significant for gender (subtracting approximately 0.5 kg of FFM in girls) and ethnicity (subtracting approximately 0.5 kg in South Asians and adding approximately 0.9 kg of FFM in black African-Caribbeans). Interactions between ethnicity and height, ethnicity and weight, ethnicity and impedance and ethnicity and gender were examined (model A3); interactions between ethnicity and weight and ethnicity and impedance were statistically significant, meaning that the regression slopes for weight and impedance varied significantly by ethnic group, while the interactions between ethnicity and height were not statistically significant. In addition, the effect of ethnicity on FFM was not appreciably modified by gender (model A3). Compared to white Europeans, the regression slope for weight was steeper among black African-Caribbeans and the regression slope for impedance was steeper among South Asians. Interactions with gender were also fitted (model A4); gender interactions with height, weight and impedance were not statistically significant therefore regression slopes for height, weight and impedance were not significantly affected by gender (model A5). Although the main effect term for black African-Caribbeans was not statistically significant in model A4, this term was highly statistically significant in the basic model (model A1) and cannot be interpreted in isolation in the presence of interaction terms with black African-Caribbean ethnicity [32]. The model of best fit selected using Akaike information criteria was model A4 which allowed for overall differences between boys and girls and ethnic groups and ethnic-specific terms for impedance and weight but only a main effect for height. This model minimised the Akaike information criterion (AIC = 2714) without appreciably decreasing the proportion of FFM variance explained (r^2^ = 0.94).

#### Type B equation: FFM = linear combination (height^2^/impedance)

Type B model coefficients are shown in Table 2. A generic model was fitted, with a term for height^2^/impedance (model B1). A problem with this equation was that 10 subjects (∼1%) had a derived FFM higher than their weight and were therefore invalid and excluded from the analysis. Intercept terms for ethnicity and gender were added (model B2); the ethnicity term was statistically significant, adding approximately 1.5 kg for black African-Caribbeans and 0.4 kg for South Asians, though the gender term was not. Interactions between ethnicity and height^2^/impedance and gender and height^2^/impedance were also fitted (model B3). The regression slopes differed by gender (steeper in girls compared to boys) and by ethnicity (steeper in South Asians compared to white Europeans). There were also significant two-way interactions between ethnicity and gender, meaning that the effect of ethnicity was modified by gender, and three-way interactions between ethnicity, gender and height^2^/impedance were added (model B4). The inclusion of three-way interactions means that the two-way interaction terms involving these variables are difficult to interpret in isolation but must be included in the model. Using this approach, model B4 minimised the Akaike information criterion (AIC = 3603) and maximised the proportion of FFM variance explained (r^2^ = 0.82).

#### Type C equation: FFM = linear combination (height^2^/impedance+weight)

Type C model coefficients are shown in Table 3; a generic model was fitted, with terms for height^2^/impedance and weight (model C1). This model was similar to the type B model except for the addition of weight. Intercept terms for ethnicity and gender were added (model C2); which were both statistically significant, adding approximately 1.0 kg for black African-Caribbeans, subtracting approximately 0.5 kg for South Asians and subtracting approximately 0.6 kg for females. As in type B models, interactions between ethnicity and height^2^/impedance and gender and height^2^/impedance were statistically significant (model C3), meaning that the regression slope for height^2^/impedance differed by ethnicity and by gender. In addition, the interaction between ethnicity and weight was statistically significant, with the regression slope being steeper in black African-Caribbeans compared to white Europeans but not significant for gender and weight (model C3). As in type B models, there were also significant two-way interactions between ethnicity and gender and three-way interactions between ethnicity, gender and height^2^/impedance were added (model C4). Using this approach, model C4 minimised the Akaike information criterion (AIC = 2888) and maximised the proportion of FFM variance explained (r^2^ = 0.92). Although the main effect term for South Asians was not statistically significant in model C4, this term was highly statistically significant in the basic model (model A1) and cannot be interpreted on its own in model C4 in the presence of interaction terms with South Asian ethnicity [32].

Overall, the best models were those of types A and C. The best individual model was A4 (Table 1), which had the lowest AIC and a slightly higher proportion of variance in FFM explained by the covariates than the next best model, C4 (Table 3). Further analyses are therefore based on the two optimal equations (A4, C4), both ethnic- and gender-specific, and their generic counterparts (A1, C1). The ethnic- and gender-specific optimal equations (A4 and C4) are shown in full in supporting information file Text S1. Type B models performed less well than either type A or type C models, with lower proportions of variance explained and higher AIC, as well as yielding infeasible values; therefore type B models will not be included in subsequent analyses.

### Bias in prediction of fat free mass in different ethnic groups using generic and ethnic and gender specific equations

**Figure 1:**
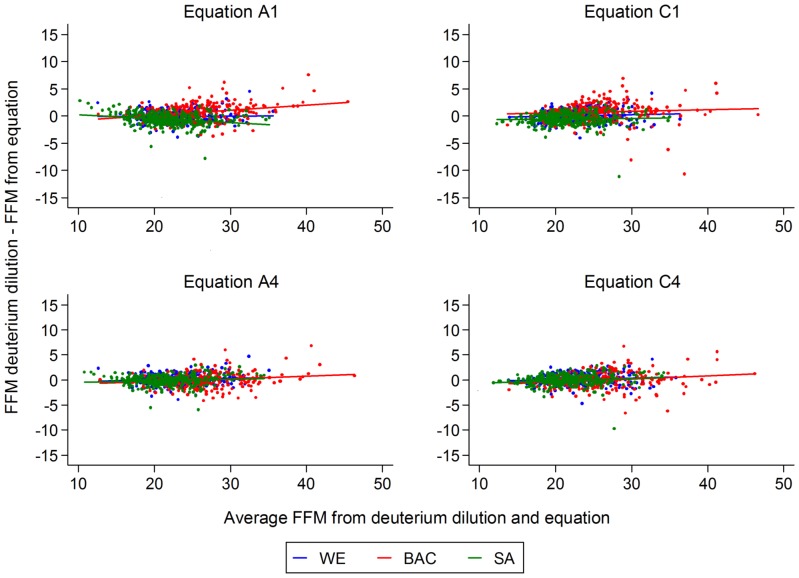
Bland-Altman plots for equations for deriving fat free mass from bioelectrical impedance analysis in ABCC Study by ethnicity. Equation A1, fat free mass = height+weight+Z (generic model); Equation A4, fat free mass = height+weight+Z (ethnic- and gender-specific model); Equation C1, fat free mass = height^2^/Z+weight (generic model); Equation C4, fat free mass = height^2^/Z+weight (ethnic- and gender-specific model). Abbreviations: FFM, fat free mass; WE, white European; BAC, black African-Caribbean; SA, South Asian; Z, impedance.

The mean difference between FFM from deuterium dilution and FFM from each predictive equation for all ethnic groups combined was closest to zero using equation A4 (mean difference, −0.01 kg), where the 95% reference range for the difference was −2.3 to 2.3 kg (supporting information file Table S2). The 95% reference ranges for the generic equations A1 and C1 were wider than the ethnic- and gender-specific equations A4 and C4. Mean levels of FFM, FM and FMI for each ethnic group, estimated by deuterium dilution and derived from BIA using prediction equations, are shown in supporting information file Table S3. Absolute levels of FFM were overestimated by approximately 0.6 kg in South Asians and underestimated by approximately 0.7 kg in black African-Caribbeans using generic equations A1 and C1. FFM was more accurately predicted among South Asians and black African-Caribbeans using ethnic- and gender-specific equations A4 and C4; there was little difference between the equations in prediction of FFM among white Europeans. Bland-Altman analyses examined the extent of bias in FFM assessment from BIA at different mean FFM levels, both in generic equations (models A1 and C1) and in ethnic- and gender-specific equations (models A4 and C4). Bland-Altman plots, showing bias in prediction of FFM by ethnicity, are presented in Figure 1. For the equations which did not take ethnicity and gender into account (equations A1 and C1 in Figure 1) there were significant differences between ethnic groups in intercepts for equations A1 and C1 (p<0.0001) and regression slopes for equations A1 only (p<0.0001). However, for both ethnic- and gender-specific equations (equations A4 and C4 in Figure 1), there were no ethnic differences in intercepts for equation A4 (p = 0.06), only slight ethnic differences in intercepts for equation C4 (p = 0.03) and no ethnic differences in regression slopes (both p>0.05); all regression slopes were close to zero, i.e. horizontal.

### Comparison of ethnic differences in fat free mass and fat mass using generic and ethnic and gender specific equations

Ethnic differences in FFM (kilograms) are shown using both generic equations (A1 and C1) and ethnic- and gender-specific equations (A4 and C4) for the ABCC Study population (Table 4) and for the CHASE Study population (Table 5). Ethnic differences in body fat outcomes including FM (derived from weight minus FFM) and FMI (FM/height^5^) are also shown in Tables 4 and 5, expressed as percentage differences due to the log transformation of these variables. Ethnic differences in FMI for all four equations and sum of skinfolds index are also shown in supporting information file Figure S1. For the ABCC Study, deuterium dilution estimates provide a reference point for ethnic differences in all outcomes, with sum of skinfolds index provided as an independent height-standardized marker of body fat. For the CHASE Study, sum of skinfolds index again provides an independent height-standardized marker of body fat. Differences in sum of skinfolds index are also expressed as percentages.

**Table 4:** Comparison of ethnic differences in body composition using different equations for deriving fat free mass in ABCC Study data.

		South Asian - White European			Black African-Caribbean - White European		
ABCC Study data (N = 814)	Equation	Difference*	(95% CI)	p (diff)	Difference*	(95% CI)	p (diff)
Fat free mass (kg)	Deuterium dilution	−1.43	(−2.03, −0.82)	<0.0001	3.15	(2.50, 3.79)	<0.0001
	A1: HT, WT, Z	−0.90	(−1.52, −0.28)	0.005	2.42	(1.76, 3.08)	<0.0001
	A4: HT, WT, Z Ethnicity and gender specific	−1.23	(−1.83, −0.64)	<0.0001	3.23	(2.60, 3.86)	<0.0001
	C1: HT^2^/Z + WT	−0.88	(−1.50, −0.26)	0.01	2.42	(1.76, 3.08)	<0.0001
	C4: HT^2^/Z + WT Ethnicity and gender specific	−1.28	(−1.87, −0.68)	<0.0001	3.26	(2.63, 3.90)	<0.0001
Fat mass (kg)*	Deuterium dilution	19.51	(10.51, 29.25)	<0.0001	19.77	(10.20, 30.17)	<0.0001
	A1: HT, WT, Z	14.86	(6.66, 23.69)	<0.001	29.88	(20.05, 40.51)	<0.0001
	A4: HT, WT, Z Ethnicity and gender specific	18.59	(9.99, 27.87)	<0.0001	19.80	(10.59, 29.77)	<0.0001
	C1: HT^2^/Z + WT	13.95	(5.46, 23.14)	<0.001	29.32	(19.09, 40.42)	<0.0001
	C4: HT^2^/Z + WT Ethnicity and gender specific	19.40	(10.40, 29.15)	<0.0001	18.60	(9.12, 28.91)	<0.0001
Fat mass index (kg/m^5^)*	Deuterium dilution	20.74	(12.79, 29.26)	<0.0001	1.89	(−5.23, 9.55)	0.61
	A1: HT, WT, Z	15.97	(8.48, 23.97)	<0.0001	10.80	(3.21, 18.95)	0.005
	A4: HT, WT, Z Ethnicity and gender specific	19.73	(12.03, 27.95)	<0.0001	2.25	(−4.72, 9.74)	0.54
	C1: HT^2^/Z + WT	14.96	(7.62, 22.81)	<0.0001	10.37	(2.89, 18.40)	0.01
	C4: HT^2^/Z + WT Ethnicity and gender specific	20.45	(12.82, 28.60)	<0.0001	1.24	(−5.56, 8.53)	0.73
Sum of skinfolds index (mm/m^3^)*		19.67	(11.65, 28.26)	<0.0001	1.68	(−5.54, 9.46)	0.66

* Percentage differences shown for log transformed variables.

Abbreviations: CI, confidence interval; HT, height; WT, weight; Z, impedance.

All differences are adjusted for gender, age quartiles, observer (skinfolds only) and a random effect for school.

**Table 5:** Comparison of ethnic differences in body composition using different equations for deriving fat free mass in CHASE data.

		South Asian - White European			Black African-Caribbean - White European		
CHASE data (N = 4425)	Equation	Difference*	(95% CI)	p (diff)	Difference*	(95% CI)	p (diff)
Fat free mass (kg)	A1: HT, WT, Z	−1.13	(−1.46, −0.80)	<0.0001	1.74	(1.42, 2.05)	<0.0001
	A4: HT, WT, Z Ethnicity and gender specific	−1.39	(−1.70, −1.08)	<0.0001	2.78	(2.48, 3.08)	<0.0001
	C1: HT^2^/Z+WT	−1.12	(−1.45, −0.78)	<0.0001	1.69	(1.36, 2.01)	<0.0001
	C4: HT^2^/Z+WT Ethnicity and gender specific	−1.46	(−1.78, −1.13)	<0.0001	2.78	(2.47, 3.09)	<0.0001
Fat mass (kg)*	A1: HT, WT, Z	2.59	(−0.75, 6.04)	0.13	15.32	(11.65, 19.10)	<0.0001
	A4: HT, WT, Z Ethnicity and gender specific	4.84	(1.40, 8.39)	0.01	5.89	(2.49, 9.41)	<0.001
	C1: HT^2^/Z+WT	1.92	(−1.56, 5.52)	0.28	15.31	(11.48, 19.27)	<0.0001
	C4: HT^2^/Z+WT Ethnicity and gender specific	5.33	(1.69, 9.10)	0.004	6.21	(2.63, 9.92)	<0.001
Fat mass index (kg/m^5^)*	A1: HT, WT, Z	4.40	(1.45, 7.42)	0.00	0.42	(−2.35, 3.27)	0.77
	A4: HT, WT, Z Ethnicity and gender specific	6.70	(3.73, 9.76)	<0.0001	−7.76	(−10.29, −5.16)	<0.0001
	C1: HT^2^/Z+WT	3.70	(0.71, 6.77)	0.01	0.33	(−2.47, 3.22)	0.82
	C4: HT^2^/Z+WT Ethnicity and gender specific	7.18	(4.10, 10.34)	<0.0001	−7.55	(−10.14, −4.90)	<0.0001
Sum of skinfolds index (mm/Height^3^)*		6.25	(2.67, 9.95)	<0.001	−10.43	(−13.38, −7.38)	<0.0001

* Percentage differences shown for log transformed variables.

Abbreviations: CI, confidence interval; HT, height; WT, weight; Z, impedance.

Adjusted for gender, age quartiles, observer (skinfolds only) and a random effect for school.

#### ABCC Study (Table 4)

The direction of ethnic differences in FFM estimated by deuterium dilution (lower levels in South Asians and higher levels in black African-Caribbeans compared to white Europeans) was correctly defined by all prediction equations, though estimates from ethnic- and gender-specific equations A4 and C4 were in closer agreement with deuterium estimates than those for generic equations A1 and C1. The use of generic equations underestimated the South Asian-white European difference in FFM by 0.53 kg and by 0.55 kg respectively for equations A1 and C1. The use of generic equations underestimated the black African-Caribbean-white European difference in FFM by 0.73 kg for both equations A1 and C1. Proportional ethnic differences in FMI from deuterium dilution were very similar to ethnic differences in sum of skinfolds index, showing markedly higher levels of body fat among South Asians and similar levels among black African-Caribbeans compared to white Europeans. The higher FMI levels in South Asians were closely reflected by ethnic- and gender-specific equations A4 and C4 but were underestimated by generic equations (by 4.8% and 5.8% respectively for equations A1 and C1). Among black African-Caribbeans, the similar FMI levels were closely reflected by ethnic- and gender-specific equations A4 and C4 but were overestimated by generic equations A1 and C1 (by 8.9% and 8.5% respectively respectively). Ethnic differences in FM (higher both in South Asians and in black African-Caribbeans) were again more accurately estimated by ethnic- and gender-specific equations A4 and C4 rather than generic equations A1 and C1, which underestimated the positive South Asian-white European FM difference (by 4.7% and 5.6% respectively) and overestimated the positive black African-Caribbean-white European FM difference (by 10.1% and 9.6% respectively). The pattern of ethnic differences was not materially affected by excluding girls who showed evidence of pubertal development (data not presented).

#### CHASE Study (Table 5)

In CHASE, the pattern of ethnic differences in FFM (lower in South Asians, higher in black African-Caribbeans) were similar to those in ABCC, with more marked ethnic differences in FFM observed using equations A4 and C4 compared with A1 and C1. Based on sum of skinfolds index, South Asian children had higher body fat levels than white Europeans, while black African-Caribbeans had lower body fat levels. The size of these percentage differences were closely matched by the percentage differences in FMI yielded by equations A4 and C4. However, the use of generic equations A1 and C1 underestimated the South Asian-white European difference in FMI, while overestimating the black African-Caribbean-white European difference, a pattern similar to that observed in the ABCC Study. For FM, the ethnic specific equations (A4 and C4) provided higher estimates of the positive FM difference between South Asians and white Europeans than the generic equations (A1 and C1), while providing lower estimates of the positive FM difference between black African-Caribbeans and white Europeans than the generic equations (A1 and C1). These patterns are very consistent with those observed in the ABCC Study (Table 4).

## Discussion

Three equation types for predicting FFM from BIA were compared. Two performed well - types A (FFM = height+weight+impedance) and C (FFM = height^2^/impedance+weight). Both benefited from the addition of terms for ethnicity and gender, including interaction terms. The use of these ethnic- and gender-specific equations (equations A4 and C4) estimated ethnic differences in body composition (particularly body fat) more accurately in the primary ABCC Study population, and more closely reflected adiposity differences based on skinfolds in the CHASE Study population. In contrast, the corresponding generic equations (equations A1 and C1) underestimated the lower levels of FFM in South Asians and the higher levels of FFM in black African-Caribbeans in the ABCC Study population. The generic equations also underestimated body fat levels among South Asians and overestimated them among black African-Caribbeans in both the ABCC Study and CHASE Study populations.

To our knowledge, this is the first study to examine the effects of using ethnic- and gender-specific equations to derive FFM from arm-leg BIA in pre-pubertal UK children on the estimation of ethnic differences in body fat. Previous studies have demonstrated a need for ethnic- and gender-specific prediction equations both in adults [33],[34] and in adolescents [20]. In adolescents, including UK Asian, black and white European 11–15 year-olds, ethnic- and gender-specific equations improved the estimation of TBW from height^2^/impedance (equivalent to model B in the present report) derived from leg-leg BIA measured with the Tanita TBF-300 body composition analyser [20]. These equations also reduced the underestimation of FM in Asians observed using the in-built Tanita equation, a finding consistent with our own observations. Studies in adults have also shown that bias in prediction of FFM and FM varies by ethnicity [33], [34]. The theoretical model of BIA treats the body like a cylinder, where electrical conductivity is proportional to cylinder length and cross-sectional area. This has led to widespread use of the ‘impedance index’, height^2^/impedance, for predicting body composition. However, in the present study the individual predictive error from this approach was large and some individuals may be predicted a FFM value exceeding weight, indicating negative FM. In our analyses, introducing weight, height and impedance separately to the model reduced this type of error, as well as improving AIC and the proportion of variance explained. This statistical approach appeared to reduce individual error, thus helping to resolve one of the principal limitations of this technique in large surveys.

Ethnic differences in the optimal equations for the prediction of FFM from BIA are likely to reflect the marked ethnic differences in body composition in children of different ethnic groups [20]. These include differences in stature (black African-Caribbean children are taller and in particular have greater leg length than white Europeans and South Asians) [13] and lean mass, particularly muscle mass, which tends to be lower among South Asians [14]. In addition, the amount and distribution of body fat varies appreciably between ethnic groups, with South Asians having a higher proportion of total fat in their abdomen [35], while black African-Caribbeans may have a lower proportion compared to white Europeans [36].

The strengths of this study include its large sample size (more than twice the size of the largest previous study [20]) with balanced numbers of children of South Asian, black African-Caribbean and white European origin, enabling reasonably precise estimation of ethnic differences in associations between FFM and covariates including impedance, height and weight or height^2^/impedance as well as detection of small intercept differences for ethnicity and gender. While the response rates were moderate and varied between ethnic groups, body composition varied widely within each ethnic group, facilitating accurate prediction across the body composition range. Although a simple ethnic group classification was used, we ensured that the South Asian study population included balanced numbers of Indian, Pakistani and Bangladeshi children and the black African-Caribbean population included both black Africans and black Caribbeans, though the study had insufficient statistical power to discriminate between these ethnic subgroups. The study examined three of the most extensively validated equation formats for deriving TBW and FFM from BIA, and used arm-leg BIA measurements, which have the merit of including both lower and upper body components. The use of measures of goodness of fit including AIC enabled robust comparisons between non-nested models and provided a basis for objective selection of preferred models. Derived equations were tested both in the study population and in a separate population, both of which had an independent assessment of body fat, based on skinfold thickness measurements. The sum of skinfolds index, though formally a marker of subcutaneous adiposity, was strongly correlated (r = 0.93) with overall adiposity, as defined by FMI from deuterium dilution. The use of two measurements of BIA in the ABCC Study but only one measurement in the CHASE Study did not materially affect the results. The use of deuterium dilution as a reference method for TBW provided a minimally invasive, accurate measurement of TBW with an error of approximately 1% [24], providing a more accurate two-compartment model for the measurement of body fat (the primary purpose of the present study) than dual-energy X-ray absorptiometry and densitometry methods [37]. Pubertal status assessment was carried out only in girls, since puberty in boys occurs at ages later than those represented in the present study [22], [23]. A minority of the girls (30%) studied showed evidence of pubertal development, however, their exclusion had no appreciable effect on the results, in agreement with the results of the earlier study in adolescents [20].

In the increasingly multi-ethnic population of the UK and many other countries, it is important to have methods for measuring body composition, particularly body fat, which are observer-independent, suitable for large scale studies and valid in all major ethnic groups. With increasing evidence that weight-for height indices, particularly body mass index, can be misleading in multi-ethnic populations [13], [38], [39], BIA provides a potentially important alternative method for body composition assessment. The results presented here highlight a need for ethnic- and gender-specific equations for predicting FFM from BIA in children. Not using ethnic- and gender-specific equations led to overestimation of body fat levels in black African-Caribbeans and underestimation in South Asians, in comparison to white Europeans. These biases are sufficiently large (particularly for black African-Caribbeans) to produce appreciable misclassification of overweight or obese individuals, if BIA-based measures of fat mass index or fat mass percentage were widely used (not the case at present), their use would also tend to underestimate the population burden of adiposity among UK South Asian children, and overestimate it among UK black African-Caribbean children. This occurred in our previous report on ethnic differences in body fat patterns in CHASE, which used the generic equation derived by Clasey et al for the estimation of FFM from BIA [13]. In this particular case, the FMI difference between South Asians and white Europeans was underestimated, but only slightly (6.6%, compared with 6.7% and 7.2% for ethnic- and gender-specific equations A4 and C4). However, the FMI difference between black African-Caribbeans and white Europeans was markedly overestimated (3.4%, compared with −7.8% and −7.6% for ethnic- and gender-specific equations A4 and C4).

The need for ethnic-specific BIA equations in pre-pubertal children of different ethnic origin, consistent with earlier reports in adolescents [20] and adults [33], [34], limits the scope for valid use of the BIA technique with generic equations in multi-ethnic populations. The present equations, based on UK South Asian, black African-Caribbean and white European children aged 8–10 years and derived using arm-leg BIA, may apply to other pre-pubertal children of similar ethnicity. However, they are unlikely to be valid in other population groups, in other age-groups [27] and in studies using different measurement procedures (e.g. leg-leg BIA). Further equations are therefore needed for deriving FFM from BIA in key ethnic groups at different ages, which will help to define the contribution of adiposity to the substantial burdens of type 2 diabetes and cardiovascular disease in ethnic minority groups and to underpin prevention.

## Conclusions

BIA is a potentially useful tool for measuring/quantifying adiposity in multi-ethnic populations. However, we have shown that ethnic- and gender-specific equations are needed for predicting FFM from BIA in pre-pubertal children of different ethnic origin. The use of such equations will help to ensure that the adiposity burdens in children from different ethnic groups are accurately defined.

## Supporting Information

Figure S1 **Comparison of ethnic differences in sum of skinfolds index and fat mass index using different equations for fat free mass from bioimpedance applied to ABCC Study and CHASE data.** Adjusted for gender, age quartiles, observer (skinfolds only) and a random effect for school. A1 = height+weight+bioimpedance equation, C1 = height^2^/bioimpedance plus weight equation, A5 = Ethnic and gender specific height+weight+bioimpedance equation, C4 = Ethnic and gender specific height^2^/bioimpedance plus weight equation. Abbreviations: SoS index, sum of skinfolds index.
(TIF)

Table S1 **Body size and composition in ABCC Study population: by gender and ethnicity.**
(DOCX)

Table S2 **Difference in fat free mass from deuterium dilution minus fat free mass derived from equations for bioelectrical impedance with 95% reference range.**
(DOCX)

Table S3 **Comparison of means using different equations for fat free mass in ABCC Study data: by ethnicity.**
(DOCX)

Text S1 **Ethnic- and gender-specific equations A4 and C4.**
(DOCX)
