# Supplementary figures and images for: Are Ethnic and Gender Specific Equations Needed to Derive Fat Free Mass from Bioelectrical Impedance in Children of South Asian, Black African-Caribbean and White European Origin? Results of the Assessment of Body Composition in Children Study
Source: PLoS One. 2013 Oct 18;8(10):e76426. doi: 10.1371/journal.pone.0076426 (PMC3799736; doi:10.1371/journal.pone.0076426)

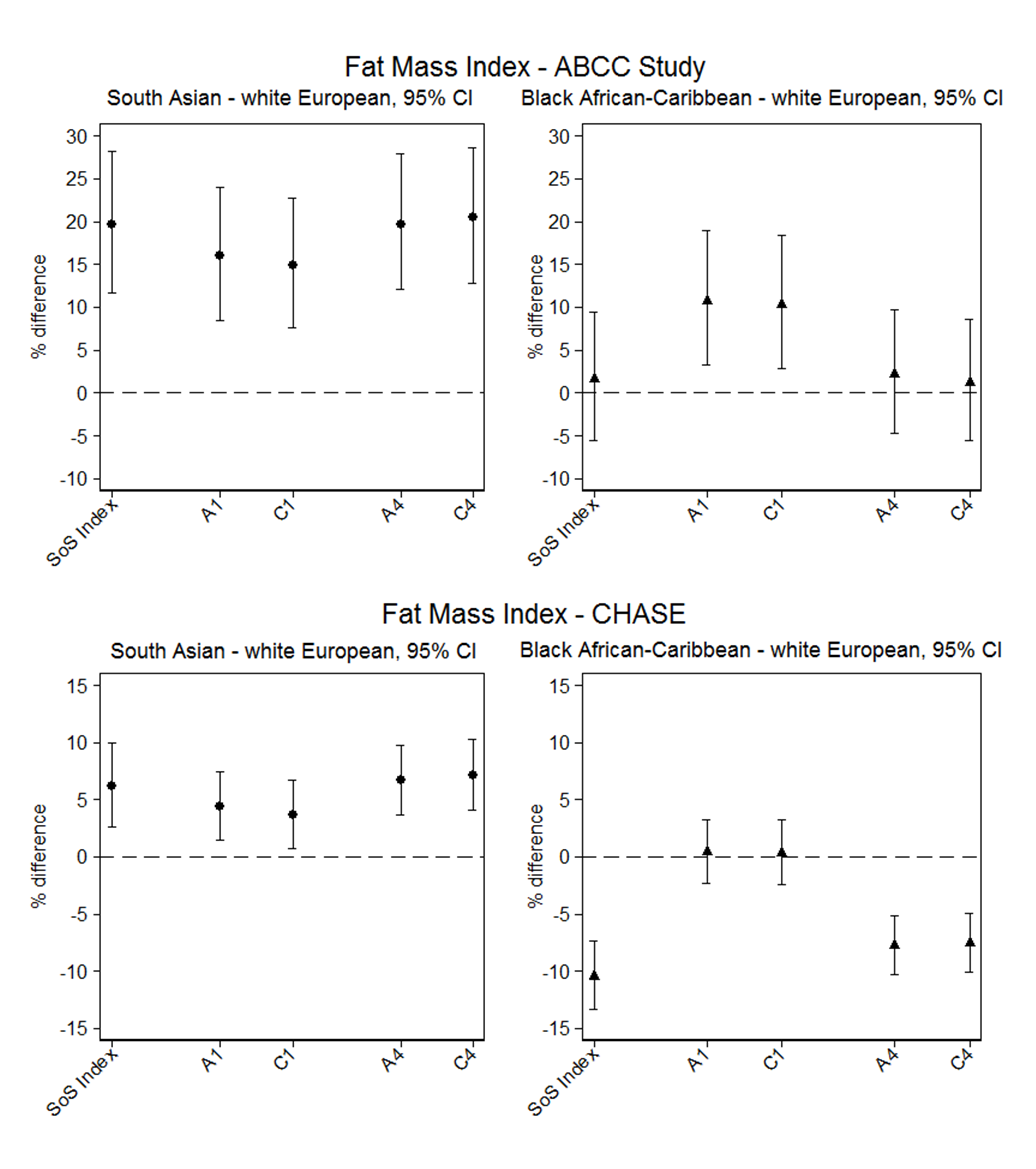

Supplement: Figure S1 — Comparison of ethnic differences in sum of skinfolds index and fat mass index using different equations for fat free mass from bioimpedance applied to ABCC Study and CHASE data. Adjusted for gender, age quartiles, observer (skinfolds only) and a random effect for school. A1 = height+weight+bioimpedance equation, C1 = height2/bioimpedance plus weight equation, A5 = Ethnic and gender specific height+weight+bioimpedance equation, C4 = Ethnic and gender specific height2/bioimpedance plus weight equation. Abbreviations: SoS index, sum of skinfolds index. (TIF) [file pone.0076426.s001.tif]
