# Supplementary material for: Are Ethnic and Gender Specific Equations Needed to Derive Fat Free Mass from Bioelectrical Impedance in Children of South Asian, Black African-Caribbean and White European Origin? Results of the Assessment of Body Composition in Children Study
Source: PLoS One. 2013 Oct 18;8(10):e76426. doi: 10.1371/journal.pone.0076426 (PMC3799736; doi:10.1371/journal.pone.0076426)
Supplement: Table S1 — Body size and composition in ABCC Study population: by gender and ethnicity. (DOCX) [file pone.0076426.s002.docx]

Supplementary Table S1: Body size and composition in ABCC Study population: by gender and ethnicity

|  | Mean (SD)* | | | | | | | | | | |  |
| --- | --- | --- | --- | --- | --- | --- | --- | --- | --- | --- | --- | --- |
|  | Boys | | Girls | |  | White European | | South Asian | | African-Caribbean | |  |
| Outcome | n = 407 | | n = 457 | | p(diff)† | n = 289 | | n = 325 | | n = 250 | | p(diff)‡ |
| Height (cm) | 136.6 | (7.3) | 135.8 | (7.3) | 0.05 | 135.0 | (7.3) | 135.0 | (7.3) | 139.2 | (7.3) | <0.0001 |
| Weight (kg)* | 33.0 | (1.3) | 32.4 | (1.3) | 0.23 | 31.0 | (1.3) | 31.5 | (1.3) | 36.2 | (1.3) | <0.0001 |
| BMI (kg/m^²^)* | 17.7 | (1.2) | 17.6 | (1.2) | 0.62 | 17.1 | (1.2) | 17.4 | (1.2) | 18.7 | (1.2) | <0.0001 |
| Sum of skinfolds (mm)* | 33.5 | (1.6) | 38.4 | (1.6) | <0.0001 | 32.6 | (1.6) | 38.7 | (1.6) | 36.6 | (1.6) | <0.0001 |
| Sum of skinfolds index (mm/m³)* | 13.3 | (1.5) | 15.4 | (1.5) | <0.0001 | 13.4 | (1.5) | 16.0 | (1.5) | 13.6 | (1.5) | <0.0001 |
| Derived from deuterium: |  | |  | |  |  | |  | |  | |  |
| Fat free mass (kg) | 24.2 | (4.5) | 22.8 | (4.5) | <0.0001 | 23.0 | (4.5) | 21.7 | (4.5) | 26.3 | (4.5) | <0.0001 |
| Fat mass (kg)* | 8.6 | (1.6) | 9.5 | (1.6) | 0.001 | 7.9 | (1.6) | 9.6 | (1.6) | 9.7 | (1.6) | <0.0001 |
| Fat mass index (kg/m^5^)* | 1.8 | (1.5) | 2.1 | (1.5) | <0.0001 | 1.8 | (1.5) | 2.2 | (1.5) | 1.8 | (1.5) | <0.0001 |

* Geometric mean and geometric SD shown for log transformed variables (95% central range is geometric mean ÷ GSD², is geometric mean x GSD²).

† p-value from a test of mean difference between genders

‡ p-value from a likelihood ratio test for hetrogeneity in association with ethnicity

Means and geometric means are adjusted for age quartiles, gender (except means by gender), ethnicity (except means by ethnicity), observer (skinfolds only) and a random effect for school. N = 814 for sum of skinfolds and sum of skindolds index.
