# Supplementary material for: Are Ethnic and Gender Specific Equations Needed to Derive Fat Free Mass from Bioelectrical Impedance in Children of South Asian, Black African-Caribbean and White European Origin? Results of the Assessment of Body Composition in Children Study
Source: PLoS One. 2013 Oct 18;8(10):e76426. doi: 10.1371/journal.pone.0076426 (PMC3799736; doi:10.1371/journal.pone.0076426)
Supplement: Table S2 — Difference in fat free mass from deuterium dilution minus fat free mass derived from equations for bioelectrical impedance with 95% reference range. (DOCX) [file pone.0076426.s003.docx]

Supplementary Table S2: Difference in fat free mass from deuterium dilution minus fat free mass derived from equations for bioelectrical impedance with 95% reference range

|  | FFM deuterium - FFM equation | |
| --- | --- | --- |
| Equation | Mean difference (95% RR) | |
| A1: HT, WT, Z | -0.04 | (-2.70, 2.62) |
| A4: HT, WT, Z Ethnicity and Gender specific | -0.01 | (-2.33, 2.31) |
| C1: HT²/Z + WT | 0.04 | (-2.86, 2.93) |
| C4: HT²/Z + WT Ethnicity and Gender specific | 0.03 | (-2.52, 2.58) |

Abbreviations: FFM, fat free mass; HT, height; WT, weight; Z, bioelectrical impedance.
