# Supplementary material for: Are Ethnic and Gender Specific Equations Needed to Derive Fat Free Mass from Bioelectrical Impedance in Children of South Asian, Black African-Caribbean and White European Origin? Results of the Assessment of Body Composition in Children Study
Source: PLoS One. 2013 Oct 18;8(10):e76426. doi: 10.1371/journal.pone.0076426 (PMC3799736; doi:10.1371/journal.pone.0076426)
Supplement: Table S3 — Comparison of means using different equations for fat free mass in ABCC Study data: by ethnicity. (DOCX) [file pone.0076426.s004.docx]

Supplementary Table S3: Comparison of means using different equations for fat free mass in ABCC Study data: by ethnicity

|  |  | Mean* (95% CI) | | | | | |
| --- | --- | --- | --- | --- | --- | --- | --- |
| ABCC Study data | Equation | White European (n=269) | | South Asian (n=307) | | African-Caribbean (n=238) | |
| Fat free mass (kg) | Deuterium dilution | 22.93 | (22.47, 23.38) | 21.50 | (21.08, 21.92) | 26.07 | (25.59, 26.55) |
|  | A1: HT, WT, Z | 22.98 | (22.51, 23.45) | 22.08 | (21.63, 22.53) | 25.40 | (24.90, 25.90) |
|  | A4: HT, WT, Z Ethnicity and Gender specific | 22.84 | (22.39, 23.29) | 21.61 | (21.18, 22.03) | 26.07 | (25.59, 26.55) |
|  | C1: HT²/Z + WT | 22.89 | (22.41, 23.36) | 22.01 | (21.56, 22.46) | 25.31 | (24.81, 25.81) |
|  | C4: HT²/Z + WT Ethnicity and Gender specific | 22.81 | (22.36, 23.26) | 21.53 | (21.10, 21.96) | 26.07 | (25.59, 26.55) |
| Fat mass (kg)* | Deuterium dilution | 7.77 | (7.29, 8.29) | 9.29 | (8.73, 9.88) | 9.31 | (8.70, 9.96) |
|  | A1: HT, WT, Z | 7.73 | (7.29, 8.20) | 8.88 | (8.40, 9.39) | 10.04 | (9.44, 10.68) |
|  | A4: HT, WT, Z Ethnicity and Gender specific | 7.82 | (7.37, 8.30) | 9.28 | (8.77, 9.82) | 9.37 | (8.80, 9.98) |
|  | C1: HT²/Z + WT | 7.79 | (7.33, 8.29) | 8.88 | (8.38, 9.41) | 10.08 | (9.45, 10.75) |
|  | C4: HT²/Z + WT Ethnicity and Gender specific | 7.82 | (7.35, 8.32) | 9.34 | (8.80, 9.90) | 9.27 | (8.69, 9.90) |
| Fat mass index (kg/m^5^)* | Deuterium dilution | 1.76 | (1.66, 1.86) | 2.12 | (2.01, 2.24) | 1.79 | (1.69, 1.90) |
|  | A1: HT, WT, Z | 1.75 | (1.66, 1.84) | 2.03 | (1.93, 2.13) | 1.93 | (1.83, 2.05) |
|  | A4: HT, WT, Z Ethnicity and Gender specific | 1.77 | (1.68, 1.86) | 2.12 | (2.01, 2.22) | 1.81 | (1.71, 1.91) |
|  | C1: HT²/Z + WT | 1.76 | (1.67, 1.86) | 2.02 | (1.92, 2.13) | 1.94 | (1.84, 2.05) |
|  | C4: HT²/Z + WT Ethnicity and Gender specific | 1.77 | (1.68, 1.86) | 2.13 | (2.02, 2.24) | 1.79 | (1.69, 1.89) |
| Sum of skinfolds index (mm/m^3^)* | | 13.34 | (12.60, 14.13) | 15.97 | (15.10, 16.88) | 13.57 | (12.77, 14.41) |

* Geometric means shown for log transformed variables

Adjusted for gender, age quartiles, observer (skinfolds only) and a random effect for school.

Abbreviations: HT, height; WT, weight; Z, bioelectrical impedance.
