# Supplementary material for: Are Ethnic and Gender Specific Equations Needed to Derive Fat Free Mass from Bioelectrical Impedance in Children of South Asian, Black African-Caribbean and White European Origin? Results of the Assessment of Body Composition in Children Study
Source: PLoS One. 2013 Oct 18;8(10):e76426. doi: 10.1371/journal.pone.0076426 (PMC3799736; doi:10.1371/journal.pone.0076426)
Supplement: Text S1 — Ethnic- and gender-specific equations A4 and C4. (DOCX) [file pone.0076426.s005.docx]

**Ethnic- and gender-specific equations A4 and C4**

Ethnic- and gender-specific equations from selected models of best fit

**Type A equations (equivalent to equation A4)**

White Europeans:

FFM(kg) = -3.919 – 0.512*sex + 0.230*HT(cm) + 0.201*WT(kg) – 0.0147*Z

Black African-Caribbeans:

FFM(kg) = -5.539 – 0.512*sex + 0.230*HT(cm) + 0.249*WT(kg) – 0.0135*Z

South Asians:

FFM(kg) = -7.844 – 0.512*sex + 0.230*HT(cm) + 0.206*WT(kg) – 0.0102*Z

**Type C equations (equivalent to equation C4)**

White Europeans:

FFM(kg) = 4.138 – 0.393*sex + 0.461*HT(cm)²/Z – 0.003*sex*HT(cm)²/Z

+ 0.214*WT(kg)

Black African-Caribbeans:

FFM(kg) = 8.376 – 3.902*sex + 0.245* HT(cm)²/Z + 0.104*sex*HT(cm)²/Z

+ 0.302*WT(kg)

South Asians:

FFM(kg) = 5.174 – 2.503*sex + 0.398* HT(cm)²/Z + 0.091*sex*HT(cm)²/Z

+ 0.218*WT(kg)

Where female = 1, male = 0.

Abbreviation: FFM, fat free mass; HT, height; WT, weight; Z, impedance.
